# Physicochemical Characteristics and Bioactive Compounds of New Black Cherry Tomato (*Solanum lycopersicum*) Varieties Grown in Vietnam

**DOI:** 10.3390/plants10102134

**Published:** 2021-10-08

**Authors:** Ho Thi Ngan Ha, Ngo Van Tai, Nguyen Minh Thuy

**Affiliations:** 1 Department of Food Technology, Faculty of Agriculture and Natural Resources, An Giang University, Long Xuyên City 90100, Vietnam; 2 Vietnam National University Ho Chi Minh City, Ho Chi Minh City 700000, Vietnam; 3 Department of Food Technology, College of Agriculture, Can Tho University, Can Tho City 900000, Vietnam; vantai@ctu.edu.vn

**Keywords:** anthocyanin, bioactive compound, black cherry tomato, LC–MS/MS, physicochemical, *Solanum lycopersicum*

## Abstract

Some physicochemical characteristics and bioactive compounds of three varieties of black cherry tomato (Indigo Rose, OG, F1:001) grown in Vietnam were investigated. The results showed that the two varieties OG and F1:001 have roughly the same size with weight, height, diameter, geometric diameter and surface area ranging from 21.62 to 22.25 g, 25.69 to 26.40 mm, 24.46 to 25.11 mm, 24.86 to 25.53 mm and 19.47 to 20.51 cm^2^, respectively. Meanwhile, the Indigo variety is twice as large with the corresponding parameters as 45.2 g, 48.03 mm, 55.18 mm, 52.69 mm and 87.20 cm^2^. All three varieties are in a spherical shape with sphericity and aspect ratios ranging from 96.72 to 109.69% and 0.951 to 1.149, respectively. The variety of OG contained higher levels of bioactive compounds, especially anthocyanin, not only in the skin but also in the outer tissue. Six anthocyanin compounds were identified in the two varieties of OG and Indigo Rose while only four anthocyanin compounds were found in the variety of F1:001. Among them, two new compounds (delphinidin-3-(p-coumaroyl)-glucoside and delphinidin-3-(p-coumaroyl)-glucoside-arabinoside) were discovered in all three varieties. The finding of this study will be a basis for consumers to better understand the nutritious properties of black cherry tomatoes grown in Vietnam, thereby promoting the need to grow and consume this beneficial fruit. The study also provides the important physicochemical parameters of black cherry tomatoes, which are the initial basis for fruit preservation and processing.

## 1. Introduction

Black cherry tomatoes (*Solanum lycopersicum* L.) are tomatoes with a purplish-brown color in their skin [1]. They have just grown in Vietnam in recent years and received great attention from consumers. Lycopene is the most abundant (about 80–90% of total carotenoid content) and the highest antioxidant natural carotenoid found in tomatoes [2]. Epidemiological studies have shown that lycopene, a red pigment, has the potential to reduce the risk of chronic diseases, most notably prostate cancer [3]. This red pigment also plays an important role in the prevention of cardiovascular disease [4]. Min and Min [5] observed that consuming a large amount of lycopene-rich foods helped to lower the risk of mortality from Alzheimer’s disease in adults. Kaur et al. [6] also found that lycopene was beneficial in the treatment of Parkinson’s disease and other neurological abnormalities by protecting against oxidative stress. In addition to carotenoids, tomatoes are good for other antioxidant compounds such as phenolics and ascorbic acid, which also inhibit reactive oxygen species causing many dangerous diseases [7]. Notably, the content of phenolic compounds and carotenoid pigments, particularly lycopene, was found to be higher in black cherry tomatoes than in some red tomato varieties [8]. Especially, black cherry tomatoes also can produce a phytochemical, anthocyanin, predominantly in the skin and outer tissue, unlike other tomatoes [9]. Anthocyanins have been proven to be associated with many health benefits, include enhancing vision, reducing cancer cell proliferation and inhibiting tumor formation, protecting against cardiovascular disease, supporting obesity and diabetes prevention, enhancing memory and preventing age-related decline in neural function, together with reducing inflammation [10]. The biological effects of anthocyanins are also due to their antioxidant activity [11].

Although black cherry tomatoes contain many bioactive compounds, research on them remains very limited, especially in Vietnam. Furthermore, consumers are still skeptical about the anthocyanin component in black cherry tomatoes because some studies in the world showed that several tomato varieties have a purple-black color as a result of mutations affecting the chlorophyll breakdown but are not related to anthocyanin production [12]. For those reasons, most of the black cherry tomatoes are grown in Vietnam gardens mainly for sightseeing and retail. This wastes a healthy raw material and also affects the economic benefits of farmers. Therefore, to create confidence for consumers, it is urgent to identify bioactive compounds in black cherry tomatoes grown in Vietnam. Additionally, understanding physicochemical properties is also very important for further preservation and processing in order to bring this high value biological material closer to consumers. 

## 2. Results

**Figure 1:**
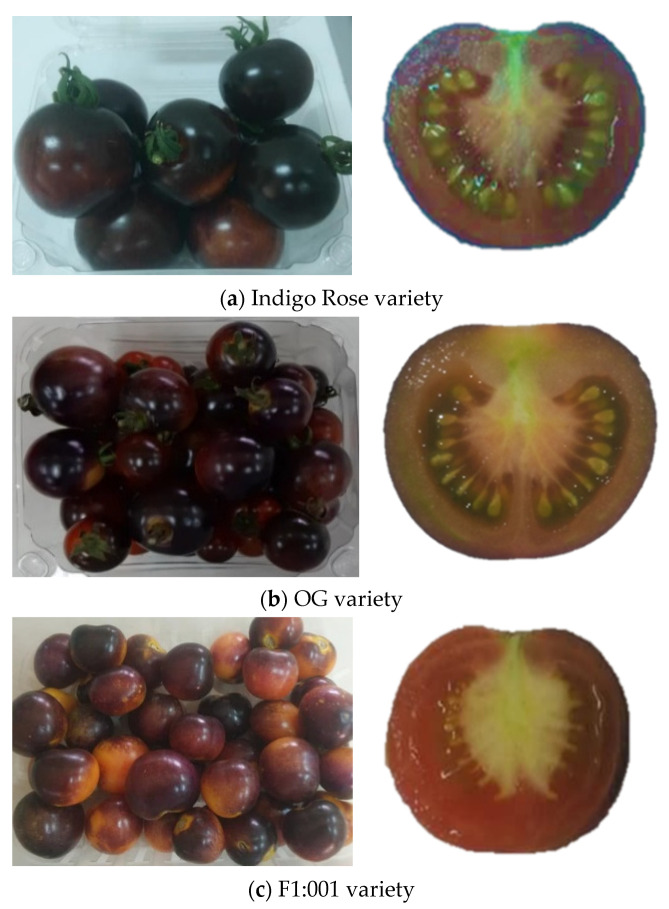
Three varieties of black cherry tomatoes grown in Vietnam. (**a**) Indigo Rose variety; (**b**) OG variety; (**c**) F1:001 variety.

Photographs of the three varieties of black cherry tomato grown in Vietnam are shown in Figure 1: Indigo Rose (a), OG (b) and F1:001 (c) at full ripening in both whole fruit and cut lengthwise. The OG variety also had a relatively stable fruit size, but the fruit weight was only about half that of the Indigo Rose variety (Table 1). Specifically, the weight of Indigo Rose, OG and F1:001 varieties fluctuated in the range 44.10–46.42 g, 20.97–23.53 g and 19.81–23.43 g, respectively, and the geometric mean diameter varied in the range 51.30–54.08 mm, 24.20–26.86 mm and 23.10–26.62 mm, respectively.

Black cherry tomato varieties were not only different in color and fruit size, but also in nutritional composition (Table 2). The moisture content was highest in the Indigo Rose variety (95.17%) and lowest in the OG variety (93.18%). In contrast, the content of two chemical components, total sugar content and total acid content, were significantly higher (5.18% and 0.475%) in the OG variety than in the other two varieties, thereby indicating the highest total soluble solid content (6.13%) and the lowest pH value (4.23) in the OG variety. In all three varieties, the purple-black color was mainly concentrated in the outer skin, as shown by the anthocyanin content in the fruit peel, which reached about 12–15 times higher than that in the fruit flesh. However, for the OG variety, it was found that anthocyanin was also synthesized extensively in the outer tissue (this could be observed when the fruit was cut in half). Therefore, the anthocyanin content in the flesh of OG variety was higher and resulted in a significantly higher level of anthocyanin content in the whole fruit (4.32 mgCE/100 g) compared with Indigo Rose (3.89 mgCE/100 g) and F1:001 (3.97 mgCE/100 g). Similarly, total phenolic content was also highest in the OG variety (39.05 mgGAE/100 g). For lycopene content, it was the highest in F1:001 and OG, exhibiting no significant difference between these two varieties (42.11 and 40.12 μg/g). Meanwhile, vitamin C content was highest (66.01 mg/100 g) in the Indigo Rose variety.

**Figure 2:**
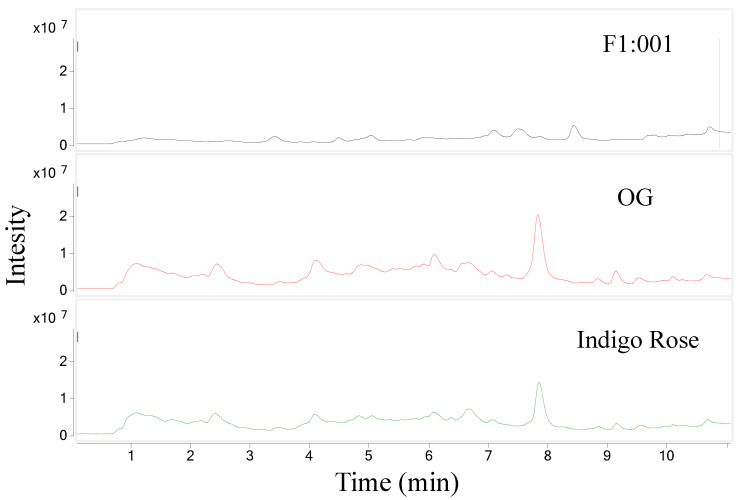
Chromatograms of [M+H]+ ions in three varieties of black cherry tomatoes.

**Figure 3:**
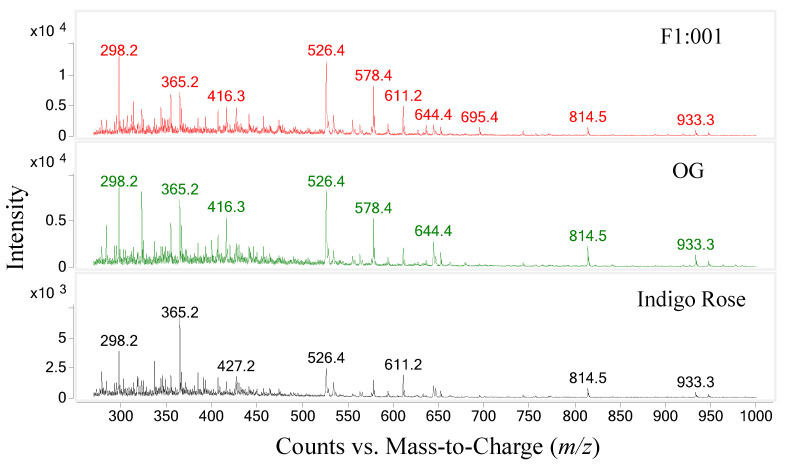
Mass spectra of [M+H]+ ions in three varieties of black cherry tomatoes.

**Figure 4:**
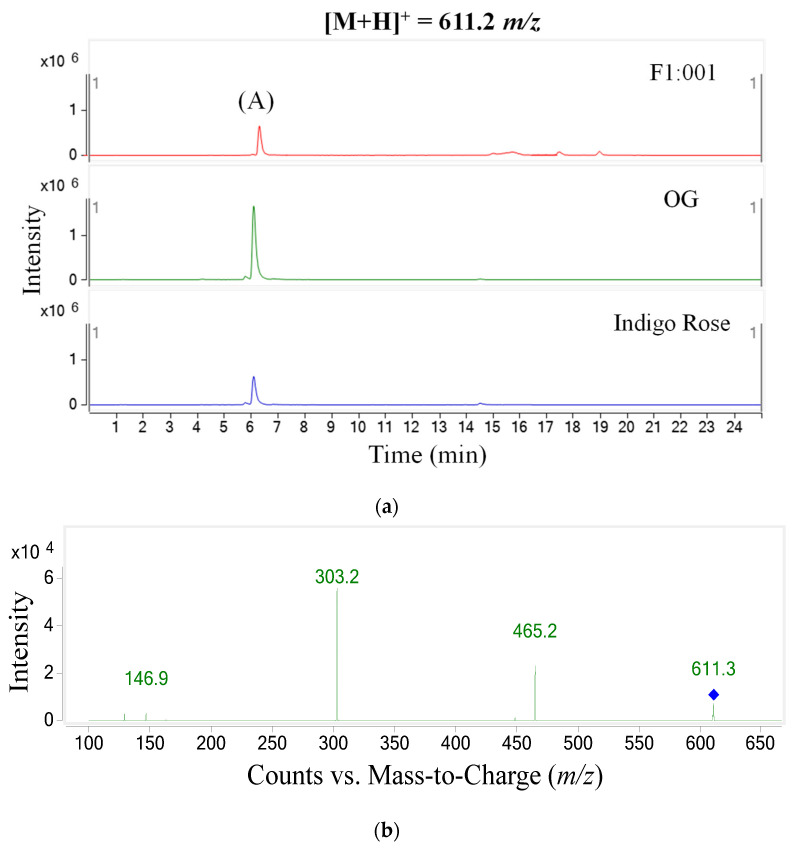
Chromatogram (**a**) and MS–MS fragments (**b**) of [M+H]+ ion with 611.2 *m*/*z* in three tomato varieties.

**Figure 5:**
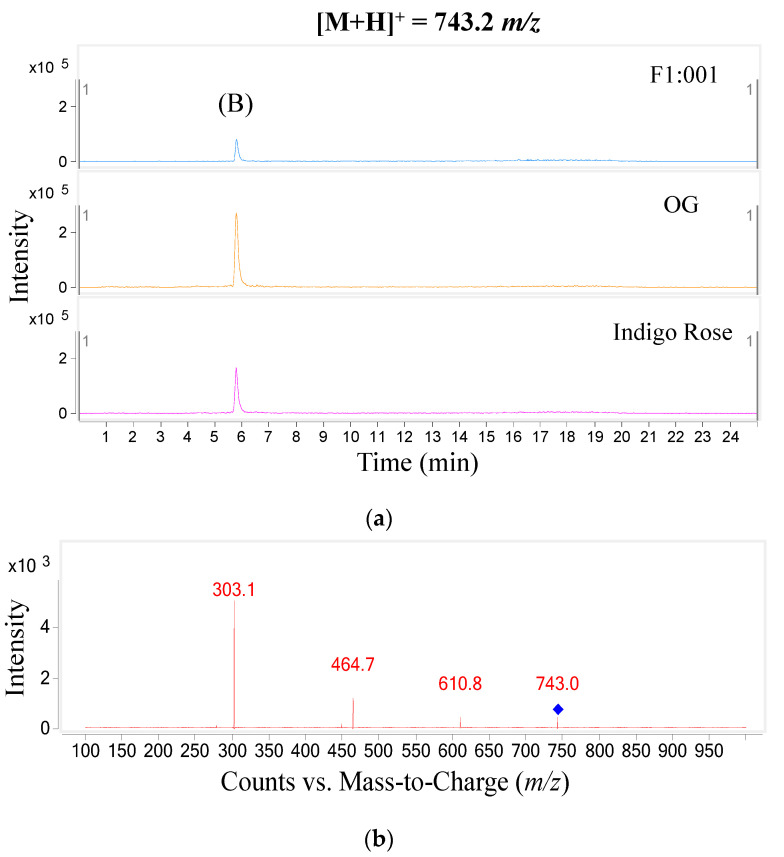
Chromatogram (**a**) and MS–MS fragments (**b**) of [M+H]+ ion with 743.2 *m*/*z* in three tomato varieties.

**Figure 6:**
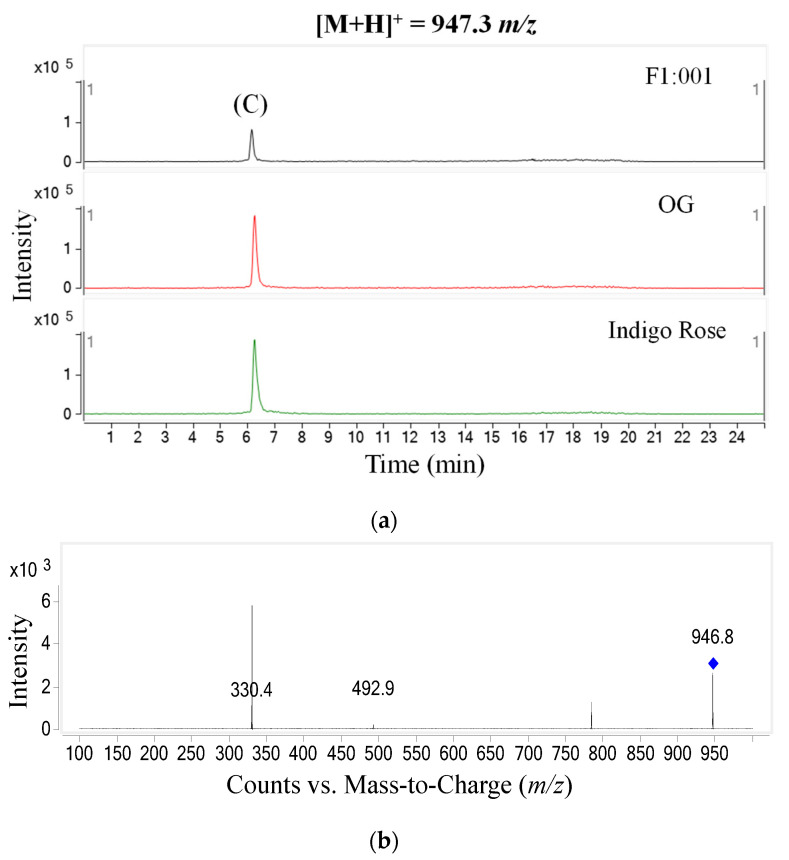
Chromatogram (**a**) and MS–MS fragments (**b**) of [M+H]+ ion with 947.3 *m*/*z* in three tomato varieties.

**Figure 7:**
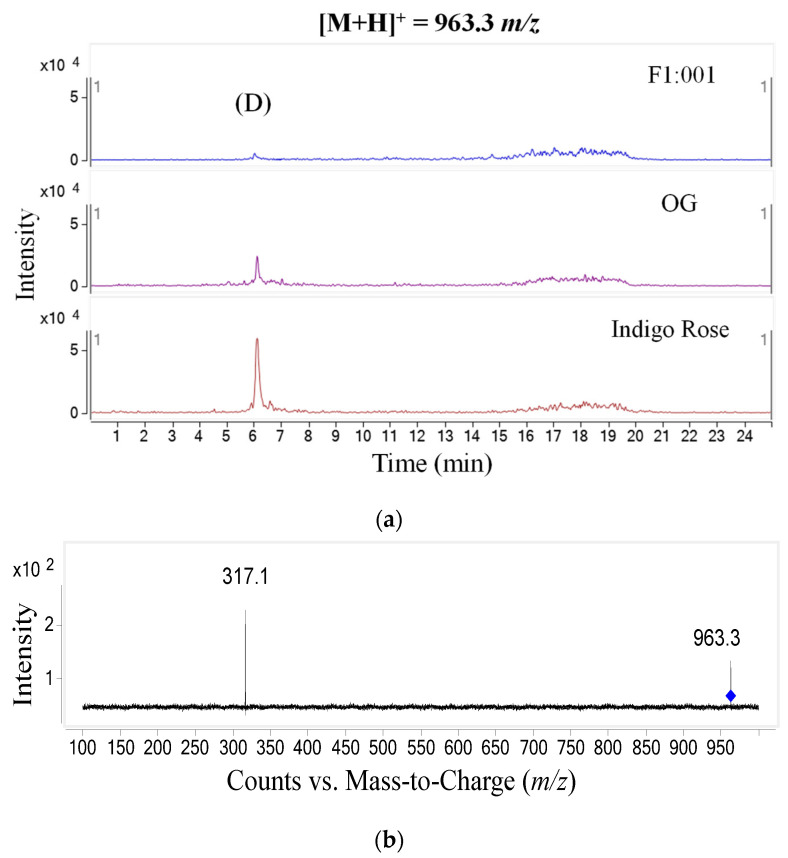
Chromatogram (**a**) and MS–MS fragments (**b**) of [M+H]+ ion with 963.3 *m*/*z* in three tomato varieties.

**Figure 8:**
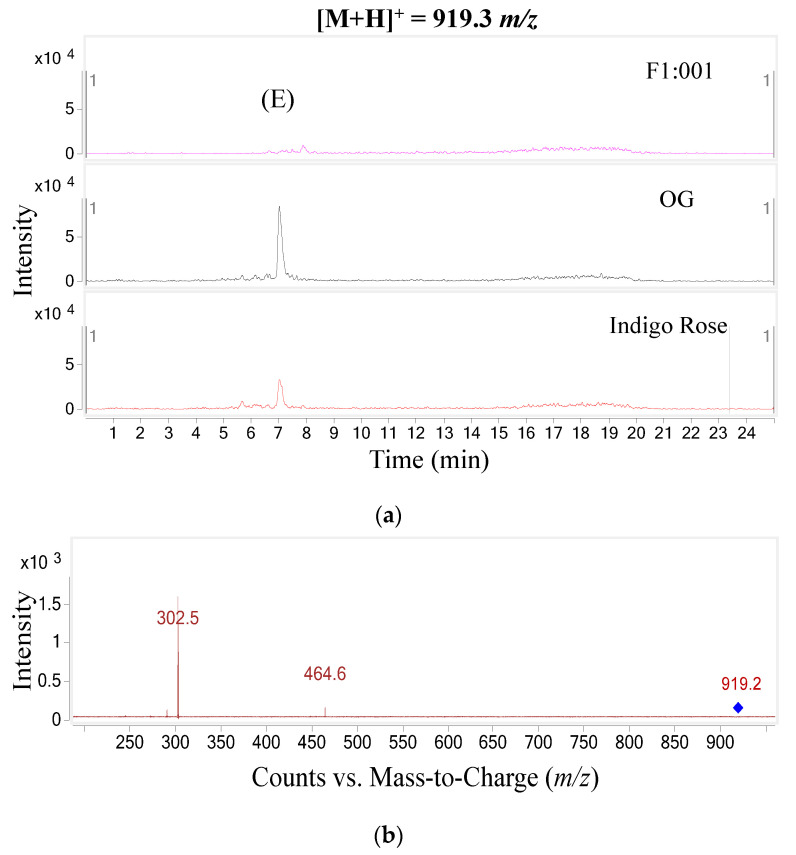
Chromatogram (**a**) and MS–MS fragments (**b**) of [M+H]+ ion with 919.3 *m*/*z* in three tomato varieties.

**Figure 9:**
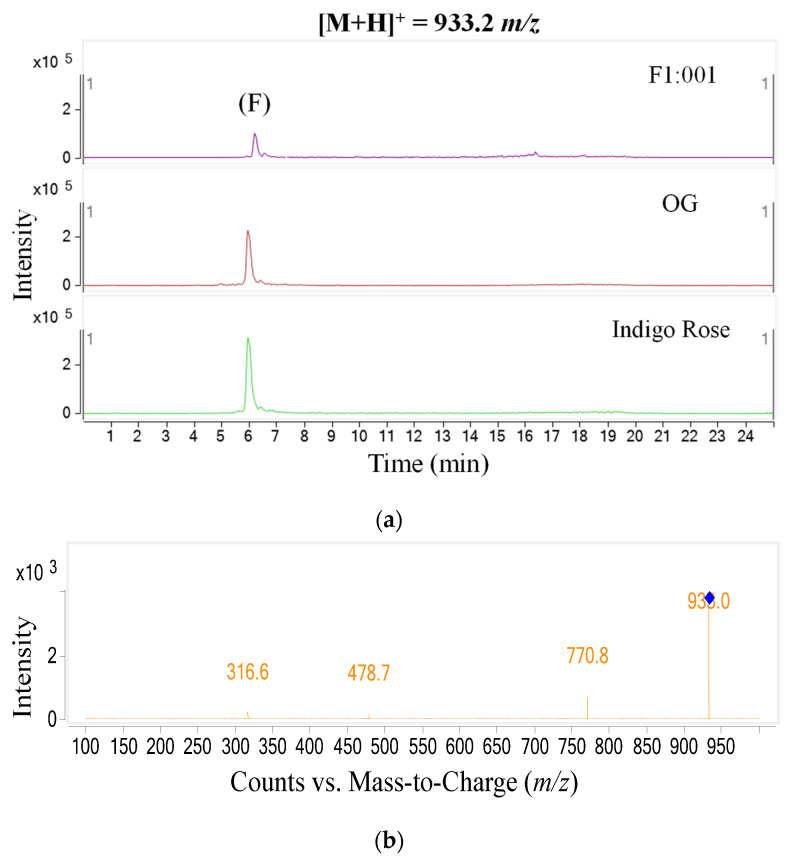
Chromatogram (**a**) and MS–MS fragments (**b**) of [M+H]+ ion with 933.2 *m*/*z* in three tomato varieties.

The study further analyzed anthocyanin composition in three black cherry tomato varieties by the LC–MS/MS method and obtained the chromatogram of [M+H]+ ions in the retention time of 10 min (Figure 2). To ensure accuracy and efficiency, UV and mass were synchronously used for anthocyanin analysis in this study. Therefore, the precursor molecular ion mass was acquired over a range from 300 to 1000 *m*/*z* (mass to charge) (Figure 3). Based on the chromatograms and mass spectra of the collected ions, combined with references to anthocyanin compounds that have been detected in a variety of fruit and vegetable materials, especially in black cherry tomatoes, the study analyzed the chromatogram of each [M+H]+ ion that was separated (Figure 4, Figure 5, Figure 6, Figure 7, Figure 8, Figure 9). As a result, four [M+H]+ ions with *m*/*z* of 611.2, 743.2, 947.3 and 933.2 were discovered in all three varieties, but two ions with *m*/*z* 963.3 and 919.3 were determined in only two varieties, OG and Indigo Rose, and in the F1:001 variety at levels below the detectable limit. The retention time of six anthocyanin peaks was spread from 5.5 to 7.5 min, approximately. The parent ions were subsequently fragmented into daughter ions and referenced sources were used to identify the specific compositions of detected anthocyanin.

The study further analyzed anthocyanin composition in the three black cherry tomato varieties by the LC–MS/MS method and obtained the chromatogram of [M+H]+ ions in the retention time of 10 min. Based on the chromatograms and mass spectra of the collected ions, combined with references to anthocyanin compounds that have been detected in a variety of fruit and vegetable materials, especially in black cherry tomatoes, the study analyzed the chromatogram of each [M+H]+ ion that was separated. Based on their structures, three different aglycones were obtained, including delphinidin (*m*/*z* 303) with three derivatives, petunidin (*m*/*z* 317) with two derivatives and malvidin (*m*/*z* 331) with one derivative (Table 3).

In the first peak (A), the *m*/*z* conversion from 611.2 to 146.9 and 303.2 indicated that the aglycone was delphinidin (*m*/*z* 303) and there was a presence of p-coumaroyl (*m*/*z* 147). In addition, a fragment with *m*/*z* 465.2 showed that there was a pyranose sugar molecule (*m*/*z* 162) bound to delphinidin. From these, the first peak was predicted to be delphinidin-3-(p-coumaroyl)-glucoside [13].

In the second peak (B), fragments similar to those at the first peak (*m*/*z* 303.1, *m*/*z* 464.7 and *m*/*z* 610.8) were obtained together with the parent ion (*m*/*z* 743.2). This evidence suggested that this anthocyanin was delphinidin-3-(p-coumaroyl)-glucoside binding to an arabinose sugar molecule (*m*/*z* 132); however, the study has not yet predicted the exact link position because a reference source was not found. 

In the third peak (C), the daughter ions obtained had *m*/*z* 330.4 (aglycone was malvidin with *m*/*z* 331); 492.9 (there was an additional pyranose sugar moiety with *m*/*z* 162 linked to malvidin); 784.7 (there was one more p-coumaroyl moiety with *m*/*z* 147 and one more pyranose moiety compared to the fragment with *m*/*z* 492.9, the two pyranose residues formed the rutinose moiety with *m*/*z* 308 linked to malvidin). Furthermore, the fragmentation of the parent ion (*m*/*z* 947.3) into the daughter ion (*m*/*z* 784.7) implied that a pyranose sugar moiety had been lost (*m*/*z* 162). From these, the anthocyanin suspected in the third peak was malvidin-3-(p-coumaroyl)-rutinoside-5-glucoside [14,15]. 

Since the fourth peak (D) (*m*/*z* 963.3) and the petunidin fragment (*m*/*z* 317) were identified in a previous study for the tomato variety of Indigo Rose [14], this anthocyanin was tentatively recognized as petunidin-3-(feruloyl)-rutinoside-5-glucoside. 

For the fifth peak (E), two fragments were obtained with *m*/*z* of 302.5 and 464.6, indicating that the aglycone was delphinidin (*m*/*z* 303) bound to a pyranose sugar moiety (delphinidin-3-pyranoside *m*/*z* 465). In addition, the *m*/*z* conversion from 919.3 to 464.6 revealed the loss of one p-coumaroyl moiety (*m*/*z* 147) and one rutinose moiety (*m*/*z* 308). Therefore, this study predicted that this anthocyanin was delphinidin-3-(p-coumaroyl)-rutinoside-5-glucoside [14,15]. 

In the sixth peak (F), the fragmentation trend was similar to that of the third peak; however, the aglycone obtained was petunidin, so the anthocyanin was tentatively identified as petunidin-3-(p-coumaroyl)-rutinoside-5-glucoside [14,15]. 

In summary, the structures of six anthocyanin compounds were identified in two varieties of black cherry tomatoes (OG and Indigo Rose), including delphinidin-3-(p-coumaroyl)-glucoside, delphinidin-3-(p-coumaroyl)-glucoside + arabinose, malvidin-3-(p-coumaroyl)-rutinoside-5-glucoside, petunidin-3-(feruloyl)-rutinoside-5-glucoside, delphinidin-3-(p-coumaroyl)-rutinoside-5-glucoside and petunidin-3-(p-coumaroyl)-rutinoside-5-glucoside.

## 3. Discussion

Three black cherry tomato cultivars grown in Vietnam were determined for physicochemical characteristics and bioactive compounds and assessed for antioxidant capacity. As shown in Figure 1, fruit weight and size are important quality parameters required for the design of a postharvest system including grading, transportation, packaging, storage, processing and kinetic monitoring of changes. Of the three varieties of black cherry tomatoes, the fruit size of the Indigo Rose variety was the largest and quite uniform. In contrast, the fruit size of the F1:001 variety was quite different; many different sizes of fruits could be harvested on the same plant. For the Indigo Rose variety, the height (longitudinal dimension from stem to blossom end) was slightly smaller than the transverse diameter (cross-sectional dimension). This was the opposite for the other two varieties. This result was consistent with the observation of Adedeji et al. [16] that tomato cultivars differ greatly in fruit shape. However, the shape indices of fruits such as sphericity (96.72% to 109.69%) and aspect ratio (0.951 to 1.149) showed that the fruit of all three black cherry tomato varieties could be considered spherical in shape. 

In the study of Mini [17], the total sugar content of the OG variety (5.18%) was also much larger than that of the red tomato variety PKM-1 (4.08%) when harvested at the red ripe stage. In contrast, the total acid content of the OG variety (0.475%) was lower than that of the PKM-1 variety (0.77%). 

In all three varieties, the purple-black color was mainly concentrated in the outer skin, as shown by the anthocyanin content in the fruit peel, which reached about 12–15 times higher than in the fruit flesh. However, for the OG variety, it was found that anthocyanin was also synthesized extensively in the outer tissue (this could be observed when the fruit was cut in half). Therefore, the anthocyanin content in the flesh of OG variety was higher and resulted in a significantly higher level of anthocyanin content in the whole fruit. Similarly, total phenolic content was also highest in the OG variety. Similar values were obtained for the bioactive compounds in the black tomato variety of V118, including lycopene 234/78 μg/g dried weight (d.w.), total phenolic 659.11 mgGAE/100 g d.w. and anthocyanin 72.31 mgCE/100 g d.w. [9]. 

Phenolic and anthocyanin are secondary plant metabolites that are also known to possess significant antioxidant activity [18]. The antioxidants are believed to partially contribute to health-promoting effects of tamarillo, including antioxidation and antioxidative stress [19], antiobesity [20] and anticancer [21], along with protection against lipid oxidation [22], which would further increase the bioactive potential for black cherry tomato as a functional ingredient. However, the contribution of the compounds to total antioxidant activity depends on the power of the antioxidant activity as well as the relative abundance of the compounds [23]. In addition to health benefits, anthocyanins have raised a growing interest in improving postharvest handling. With the presence of anthocyanins, reduced overripening and longer shelf-life have been observed [24], and this may explain the long season of black cherry tomatoes. Anthocyanins enhance the antioxidant activity of fruits by suppressing reactive oxygen species, which will subsequently slow the over ripening process [25]. By controlling the reactive oxygen species burst, anthocyanins improve fruit resistance to botrytis, thereby minimizing fungal growth [25]. Together with other phenolic compounds and due to the diversity and high content of anthocyanins, the OG variety also showed the highest antioxidant activity, as shown by the lowest IC50 value of the ability to scavenge DPPH radicals.

The structural modifications of these anthocyanin compounds were mainly glycosylation, and there was also acylation with acid p-coumaric. With the addition of hydroxyl groups and sugars and methylation, these compounds are further derived to form various anthocyanin compounds in the black cherry tomatoes examined in the current study. For example, an addition of a sugar molecule to the delphinidin-3-(p-coumaroyl)-glucoside produced delphinidin-3-(p-coumaroyl)-glucoside + arabinose, which was detected in this study (Table 3). These modifications increase the chemical stability of anthocyanins [26]. Among these, two anthocyanin compounds, namely malvidin-3-(p-coumaroyl)-rutinoside-5-glucoside and petunidin-3-(p-coumaroyl)-rutinoside-5-glucoside were found in black tomato variety of V118 [9] and four anthocyanin compounds, including malvidin-3-(p-coumaroyl)-rutinoside-5-glucoside, petunidin-3-(feruloyl)-rutinoside-5-glucoside, delphinidin-3-(p-coumaroyl)-rutinoside-5-glucoside and petunidin-3-(p-coumaroyl)-rutinoside-5-glucoside were found in the variety of Indigo Rose in a previous study by Wang et al. [14]. Two other delphinidin derivatives, delphinidin-3-(p-coumaroyl)-glucoside and delphinidin-3-(p-coumaroyl)-glucoside + arabinose, found from black cherry tomatoes have not been published in previous scientific research. Variations in the profiles of anthocyanin compounds in black cherry tomatoes between the current study and the previously reported values in the literature may be partly explained by cultivars of fruit, postharvest handling, storage period, ripeness of the fruit and extraction and analytical methods. Variations may also have been from the climate and environmental factors [18]. Current findings support the previous literature that delphinidin-based anthocyanins are the abundant structure of Solanaceous species, including pepper, tomato, eggplant and potato [24]. Delphinidin-based anthocyanins have been reported as inhibiting thrombosis and reducing vascular inflammation [27]. Additionally, by preventing keratinocyte apoptosis, it is able to protect human skin against UV-B irradiance [24].

The results obtained showed that the black cherry tomato variety OG contained high levels of bioactive compounds, especially capable of synthesizing a large amount of anthocyanin, not only concentrated in the skin but also in the flesh.

## 4. Materials and Methods

### 4.1. Experimental Design

The study conducted a preliminary survey in some areas where black cherry tomatoes were grown in Vietnam. As a result, three varieties of black cherry tomatoes (Indigo Rose, OG, F1:001) were obtained from three regions: Ho Xuan Huong Agricultural Village (Da Lat City, Lam Dong Province), Nam Long Production and Trading Facility (Vinh Long City, Vinh Long Province) and Safe Vegetable Production Team (Chau Doc City, An Giang Province). Three varieties were collected and grown in a greenhouse at Nam Long Production and Trading Facility with the same conditions of nutrition and care. The fruits were harvested when they were fully ripened. Harvest time was in the morning (before 8 a.m). After harvest, tomatoes were put into polyvinyl chloride (PVC) boxes and then into cartons to avoid damage to the fruits. Both plastic boxes and cartons were perforated to create ventilation. The fruits were then transported to the Food Technology Laboratory in Can Tho University (Can Tho City) within 1 h.

### 4.2. Analytical Methods

#### 4.2.1. Physical Characteristics

*a.*  *Fruit Weight and Size Parameters*

The fruit weight was determined by an analytical balance with 0.0001 g accuracy (PR-series, Ohaus, NJ, US). The fruit size was determined by two dimensions, namely height (*H*) and diameter (*D*), which were measured by using digital calipers with an accuracy of 0.01 mm (MC 01120028, Gaogen, China). The geometric mean diameter (*D_g_*) was calculated by Equation (1) [28].

(1) $$D_{g}(\mathrm{mm})={\left(HD^{2}\right)}^{1/3}$$

The surface area (*S_a_*) and aspect ratio (*R_a_*) of the fruit were calculated by using Equations (2) and (3), respectively [28].

(2) $$S_{a}({\mathrm{cm}}^{2})=\pi D_{g}^{2}$$

(3) $$R_{a}=\frac{D}{H}$$

The sphericity (*S_p_*), defined as the ratio of the surface area of a sphere having the same volume as that of fruit to the surface area of the fruit, was determined using Equation (4) [28].

(4) $$S_{p}(\%)=100\times \frac{D_{g}}{H}$$

*b.*  *Total soluble solids (TSS) content and pH value*

The whole tomatoes were ground in a blender (MX-GM1011, Panasonic, Japan) for 1 min and the puree obtained was analyzed for TSS content and pH. The content of TSS was measured by a refractometer (0–32%, Atago, Japan) at 20 °C. The pH value was measured with a pH meter (Edge HI2020-01, Hanna, Vietnam) at 20 °C.

#### 4.2.2. Chemical Characteristics

*a.*  *Moisture content*

The moisture content was determined by drying the sample at 105 °C to a constant weight and calculated based on the weight of the original sample and the weight of the sample after drying [29].

*b.*  *Total sugar content*

The total sugar content was determined by the colorimetric method with DNS reagent [30]. Tomato puree (3 g) was weighed into a 100 mL flask with 50 mL of distilled water and 5 mL of 36.5% HCl solution. The hydrolysis process was carried out at 60 °C for 15 min. The mixture was neutralized by the 30% NaOH solution to pH 7.0, then filled to a volume of 100 mL with distilled water and filtered through a filter paper. Next, 1 mL DNS reagent was added to the filtrate (2 mL) and placed in boiling water for 5 min. The absorbance of the mixture was read at 540 nm using a UV–Vis spectrophotometer (722N, Inesa, China). The total sugar content was determined based on the reducing sugar content derived from the standard curve.

*c.*  *Total acid content*

The total acid content was determined by the titration method [28]. Tomato puree (10 g) was shaken with neutral water for 1 h. The mixture was filled to a volume of 50 mL with neutral water and allowed to settle. Five drops of phenolphthalein were added to the supernatant (25 mL) and titrated with 0.1 N NaOH solution until the mixture had a stable light pink color. The total acid content was calculated based on the volume of NaOH solution used to titrate.

#### 4.2.3. Bioactive Compounds and Antioxidant Activity

*a.*  *Anthocyanin content*

The anthocyanin content was determined by the pH differential method of Lee et al. [31] with some modifications. Tomato puree (5 g) was filled to a volume of 50 mL with ethanol/water (1/1) solvent containing 1% HCl and extracted for 60 min. The mixture was then separated by a centrifuge at 7000× *g* for 10 min. The supernatant was diluted with two buffers of pH 1.0 and 4.5 and the absorbance read at both 520 and 700 nm versus a blank of distilled water. The anthocyanin content was calculated as cyanidin-3-glucoside equivalent (CE) based on the absorbance of two dilutions at two wavelengths.

*b.*  *Lycopene content*

The lycopene content was determined by the low volume hexane extraction method [32,33]. Tomato puree (0.6 g) was mixed with 5 mL of acetone containing 0.05% butylated hydroxytoluene, 5 mL of 95% ethanol and 10 mL of hexane in a vial and extracted for 15 min on a shaker at a speed of 180 rpm. Three mL of the mixture was then added to deionized water and shaken for 5 min. The vial was left for 5 min. The absorbance of the supernatant was read at 503 nm against a hexane blank. The lycopene content was determined based on the absorbance of the extract at 503 nm.

*c.*  *Vitamin C content*

The vitamin C content was determined by the titration method [34]. Tomato puree (10 g) was filled to a volume of 100 mL with 5% HCl solution and filtered through a filter paper. Five drops of the 1% starch solution were added to the filtrate (10 mL) and titrated with the 0.001 N KIO_3_/KI solution until a blue-black color appeared. For the control, the sample extract was replaced by the 1% HCl solution. The vitamin C content was calculated based on the volume of 0.001 N KIO_3_/KI solution used for titration of the extract and the control.

*d.*  *Total phenolic content*

The total phenolic content was determined using Folin–Ciocalteu reagent (Teixeira et al. [35]) with some modifications. Tomato puree (5 g) was filled to a volume of 50 mL with 95% ethanol and extracted for 60 min. The mixture was then separated by a centrifuge at 7000× *g* for 10 min. One milliliter of 10% Folin–Ciocalteu reagent was added to the supernatant (0.2 mL), left for 5 min and then 1.2 mL of 5% Na_2_CO_3_ solution was added. After 2 h, the absorbance was recorded at 750 nm. The total phenolic content was calculated as gallic acid equivalent (GAE) based on the content of gallic acid derived from the standard curve.

*e.*  *Antioxidant activity*

Antioxidant activity was determined using the DPPH assay of Teixeira et al. [35] with some modifications. Tomato puree (5 g) was filled to a volume of 50 mL with 95% ethanol and extracted for 60 min. The mixture was then separated by a centrifuge at 7000× *g* for 10 min. Two milliliters of DPPH solution (0.21 mM in 95% ethanol) was added to the supernatant (0.1 mL). For the control, the sample extract was replaced with 95% ethanol. The mixture was kept for 1 h before an absorbance reading at 517 nm. The percentage of DPPH free radical scavenging was calculated based on the absorbance of the control and the sample. The sample concentration (in 1 mL reaction mixture) providing 50% inhibition (IC_50_) was estimated by plotting percentages of inhibition against concentrations of the sample.

*f.*  *Anthocyanin composition*

Black cherry tomato samples were analyzed for anthocyanin composition using the liquid chromatography–mass spectrometry (LC–MS/MS) method of Stein-Chisholm [13] with some modifications. 

Sample preparation: Tomatoes (200 g) were lyophilized in a freeze dryer (Alpha-2-4 DL, Martin Christ, Germany) at −80 °C and 0.001 mbar to 3–5% moisture.

Extraction: After lyophilization, tomato powder (5 g) was extracted twice in 50 mL of extraction solvent (methanol:water, 60:40; v/v) for 60 min with ultrasonic assistance (WUC-AH, Daihan, Korea) at room temperature (23–25 °C). The mixture was then separated by a centrifuge at 16,000× *g* for 30 min. 

Purification: The supernatant (100 μL) was added to 700 μL of ice ethanol. The mixture was thoroughly mixed by a vortex for 30 s and kept at −80 °C for 60 min. The mixture was then centrifuged at 21,000× *g* for 30 min. The supernatant was filtered through a polyvinylidene fluoride syringe filter (VWR Scientific, Seattle, WA, US, diameter of 17 mm, thickness of 0.2 mm, pore size of 0.2 μm) and dried at 40 °C under vacuum condition. The solid fraction was redissolved in 6 mL of 100% methanol. Before loading, the sample was diluted in 100 μL of water. The mixture was passed through a C18 solid-phase extraction column and washed with water (6 mL), then eluted with methanol (6 mL). The methanol fraction containing anthocyanins was dried and redissolved in 100 mL.

UPLC–MS/MS: The separation of anthocyanins was performed on a Zorbax Eclipse Plus C18 column (2.1 × 50 mm; 1.8 μm, Agilent, CA, US). The mobile phase consisted of 99.9% deionized water and 0.1% formic acid (A) and 90% acetonitrile with 9.9% water and 0.1% formic acid (B). The flow rate was 0.3 mL/min and the mobile phase changed as follows: 0–5 min, 70% A, 30% B; 5–8 min, 25% A, 75% B; 8–17 min, 0% A, 100% B; 18–25 min, 100% A, 0% B. The UV absorption spectrum of anthocyanins was recorded at 535 nm. Anthocyanins were then determined using a triple quadrupole system (QQQ, 6460, Agilent, CA, US). The MS/MS fragmentation was performed at 120 V using N_2_ as carrier gas with a gas temperature of 275 °C and a gas flow rate of 8 L/min. Scanning mass spectroscopy ranged from 300 to 1000 m/z (positive charge).

### 4.3. Data Analysis 

Data analyses were carried out using the Statgraphics Centurion software (version XV (US). The significance/nonsignificance of results was determined using the one-way ANOVA and least significant difference test at the 95% confidence level (*p* = 0.05).

## 5. Conclusions

Of the three black cherry tomato varieties (Indigo Rose, OG, F1:001) grown in Vietnam, the OG variety had a small and uniform size and contained higher levels of nutritional and bioactive compounds, especially anthocyanins. A total of six anthocyanins were detected in the OG tomato variety. The results obtained confirmed the presence of anthocyanin in black cherry tomatoes along with a large amount of other bioactive compounds, thereby helping consumers and processors realize the value of this fruit. One of the original contributions of our work to the anthocyanin profile of the black cherry tomato is the identification of two compounds as delphinidin-3-(p-coumaroyl)-glucoside and delphinidin-3-(p-coumaroyl)-glucoside + arabinose, which have not been reported in the previous research literature for tomato varieties. To our knowledge, this was the first attempt to determine the composition and quantify anthocyanin in the OG black cherry tomato variety.

However, it is necessary to study more deeply and widely in order to fully detect anthocyanin compounds and correctly determine their structures in black cherry tomatoes. In addition, the content of each anthocyanin component also needs to be quantified to better understand anthocyanin stability characteristics during later storage and processing. A detailed “map” of polyphenols should be further analyzed for a more comprehensive assessment of antioxidant capacity because an antioxidant capacity and free radical scavenging power could be not only given by anthocyanin but even by other polyphenolic compounds. Furthermore, additional measures of antioxidant activity such as the ORAC test (oxygen radical absorbance capacity) should be carried out to evaluate the capacity of these tomatoes in inhibiting free oxygen radicals.

## Figures and Tables

**Table 1:** Fruit weight and size of three varieties of black cherry tomatoes.

Parameters	Varieties		
	Indigo Rose	OG	F1:001
Weight (g)	45.26 ^b^ ± 1.16	22.25 ^a^ ± 1.28	21.62 ^a^ ± 1.81
Height (mm)	48.03 ^b^ ± 1.15	26.40 ^a^ ± 1.36	25.69 ^a^ ± 1.73
Diameter (mm)	55.18 ^b^ ± 1.52	25.11 ^a^ ± 1.31	24.46 ^a^ ± 1.77
Geometric mean diameter (mm)	52.69 ^b^ ± 1.39	25.53 ^a^ ± 1.33	24.86 ^a^ ± 1.76
Sphericity (%)	109.69 ^b^ ± 2.64	96.72 ^a^ ± 0.42	96.77 ^a^ ± 3.26
Surface area (cm^2^)	87.20 ^b^ ± 2.30	20.51 ^a^ ± 1.06	19.47 ^a^ ± 1.37
Aspect ratio	1.149 ^b^ ± 0.041	0.951 ^a^ ± 0.006	0.952 ^a^ ± 0.048

Notes: The results are presented as mean ± standard deviation; Values with different superscripts within a row are significantly different at a 5% significance level (*p* < 0.05).

**Table 2:** Physicochemical, bioactive compounds and antioxidant activity of three black cherry tomato varieties.

Parameters	Varieties		
	Indigo Rose	OG	F1:001
Moisture content (%)	95.17 ^c^ ± 0.20	93.18 ^a^ ± 0.24	94.48 ^b^ ± 0.20
Total soluble solid content (%)	4.13 ^a^ ± 0.12	6.13 ^c^ ± 0.12	5.60 ^b^ ± 0.20
pH value	4.58 ^b^ ± 0.17	4.23 ^a^ ± 0.10	4.49 ^ab^ ± 0.16
Total sugar content (% *w*/*w*)	3.58 ^a^ ± 0.20	5.18 ^c^ ± 0.25	4.36 ^b^ ± 0.20
Total acid content (% *w*/*w*)	0.416 ^a^ ± 0.017	0.475 ^b^ ± 0.023	0.447 ^ab^ ± 0.024
Anthocyanin content (mgCE/100 g)			
Whole fruit	3.89 ^a^ ± 0.09	4.32 ^b^ ± 0.12	3.97 ^a^ ± 0.06
Fruit skin	19.24 ^ab^ ± 0.38	19.87 ^b^ ± 0.34	19.07 ^a^ ± 0.46
Fruit flesh	1.34 ^a^ ± 0.03	1.57 ^b^ ± 0.05	1.27 ^a^ ± 0.04
Lycopene content (μg/g)	23.81 ^a^ ± 0.84	40.12 ^b^ ± 0.78	42.11 ^b^ ± 1.67
Vitamin C content (mg/100 g)	66.01 ^c^ ± 1.20	54.43 ^a^ ± 1.38	59.06 ^b^ ± 1.19
Total phenolic content (mgGAE/100 g)	31.10 ^a^ ± 0.86	39.05 ^b^ ± 0.21	31.02 ^a^ ± 0.26
IC_50_ value (mg/mL)	3.21 ^c^ ± 0.03	2.63 ^a^ ± 0.03	2.85 ^b^ ± 0.05

Notes: The results are presented as mean ± standard deviation; Values with different superscripts within a row are significantly different at a 5% significance level (*p* < 0.05).

**Table 3:** Anthocyanins found in three varieties of black cherry tomatoes.

Peak	[M+H]+ (*m*/*z*)	Detected Fragments	Anthocyanins	Varieties
A	611.2	465.2 (delphinidin-3-pyranoside; 303.2 (delphinidin); 146.9 (p-coumaroyl)	Delphinidin-3-(p-coumaroyl)-glucoside)	Indigo Rose, OG, F1:001
B	743.2	610.8 (delphinidin-3-(p-coumaroyl-glucoside); 464.7 (delphinidin-3-pyranoside); 303.1 (delphinidin)	Delphinidin-3-(p-coumaroyl)-glucoside +arabinose	Indigo Rose, OG, F1:001
C	947.3	784.7 (malvidin-3-(p-coumaroyl)-rutinoside); 492.9 (malvidin-3-pyranoside); 330.4 (malvidin)	Malvidin-3-(p-coumaroyl)-rutinoside-5- glucoside	Indigo Rose, OG, F1:001
D	963.3	317 (petunidin)	Petunidin-3-(feruloyl)-rutinoside- 5-glucoside	Indigo Rose, OG
E	919.3	464.6 (delphinidin-3-pyranoside); 302.5 (delphinidin)	Delphinidin-3-(p-coumaroyl)-rutinoside- 5-glucoside	Indigo Rose, OG
F	933.2	770.8 (petunidin-3-(p-coumaroyl)-rutinoside); 478.7 (petunidin-3-pyranoside); 316.6 (petunidin)	Petunidin-3-(p-coumaroyl)-rutinoside- 5-glucoside	Indigo Rose, OG, F1:001

## Data Availability

Data is contained within the article.
